# Analysis of urban–rural differences in cognitive function among empty nest older adult in China based on Blinder-Oaxaca decomposition

**DOI:** 10.3389/fpubh.2025.1577541

**Published:** 2025-07-30

**Authors:** Xiao Pan, Gui-Ning Zhang, Li-Chong Lai, Li-Yan Zhang, Hui-Qiao Huang

**Affiliations:** ^1^Ear, Nose, Throat, Head and Neck Surgery, The Second Affliated Hospital of Guangxi Medical University, Nanning, China; ^2^Scientific Research Department, The Second Affliated Hospital of Guangxi Medical University, Nanning, China; ^3^Department of Nursing, The Second Affliated Hospital of Guangxi Medical University, Nanning, China; ^4^Party Committee Office, The Second Affliated Hospital of Guangxi Medical University, Nanning, China

**Keywords:** empty nest older adult, the risk of cognitive impairment, urban and rural, difference analysis, Blinder-Oaxaca decomposition

## Abstract

**Objective:**

This study aims to explore the cognitive status and urban–rural differences of empty nest older adult in China, analyze in depth the possible reasons for these differences, and provide reference for developing targeted prevention strategies for the risk of cognitive impairment.

**Methods:**

A cross-sectional survey was conducted on empty nest older adult people from 35 cities and rural areas in 14 regions of Guangxi, China to evaluate their chronic disease prevalence, anxiety, depression, and cognitive status. The influencing factors and sensitivity of cognitive function impairment in empty nest older adult people in urban and rural areas were analyzed, and the Oaxaca Blinder decomposition method was used to analyze the urban–rural differences in cognitive function of empty nest older adult people.

**Results:**

A total of 2083 empty nest older adult people were included, with a prevalence of the risk of cognitive impairment of 30.24%. Among them, the proportion of the risk of cognitive impairment was 33.33% (362/1086) in rural empty-nest older adult, higher than 26.88% (268/997) in urban empty-nest older adult. Older age, lower education level, and depression were common risk factors for cognitive impairment in both urban and rural empty-nest older adult (*p* < 0.05). Notably, rural empty-nest older adult showed sensitivity to the number of chronic illnesses and cervical and lumbar spondylosis. Among the differences in cognitive function among empty-nest older adult, 47.64% were related to the place of residence itself. Individual characteristic differences between urban and rural empty-nest older adult in education level (44.09%), number of chronic illnesses (27.74%), depression (15.75%), osteoporosis (10.79%), and age (6.19%) exacerbated the cognitive function differences.

**Conclusion:**

The proportion of the risk of cognitive impairment among empty nest older adult in rural areas is higher than that in urban areas, and education level is the most important factor affecting the difference in cognitive function between urban and rural areas. It is suggested to improve health education in rural areas, narrow the urban–rural gap in cognitive function of empty nest older adult, and promote fairness in medical service supply.

## 1 Background

The issue of global population aging is becoming increasingly severe, and aging and its related symptoms have become hot topics for in-depth research by scholars at home and abroad (1). Cognitive impairment is an important manifestation of brain function decline in humans, encompassing multiple aspects such as memory, thinking, language, attention, and judgment. The cognitive status of the older adult not only directly affects their competence in daily work and quality of life but also leads to a reduction in life expectancy, imposing a heavy burden on caregivers and exerting tremendous pressure on social medical resources (2, 3). Currently, approximately 50 million older adult people worldwide suffer from cognitive dysfunction, with projections reaching 152 million by 2050, and China alone expected to have 28.98 million cases (4).

Changes in cognitive function among the older adult are closely related to family structure and social support (5). Empty nest older adult refer to those who live without their children or whose children do not reside with them. Currently, China is facing a surge in the number of empty nest older adult, particularly since the reform and opening-up period, when large-scale migration of young labor force to cities and overseas has exacerbated population aging and “empty nestification” in rural areas, becoming a significant feature of changes in rural family structure (6). Empty nest older adult, especially those who are frail and older adult, face more obstacles in accessing healthcare, and their care issues have increasingly become the focus of attention from all sectors of society (7). Against the backdrop of changes in family structure, the cognitive development of the older adult is influenced by multiple factors such as sociodemographic characteristics, disease status, and formal (such as government support) and informal (such as family care) support networks (8), showing a non-linear downward trend with age, and this downward trend is significantly different between urban and rural areas (9). At the same time, we found that in the context of urban–rural differences, although urban empty nesters may face the loneliness caused by the lack of children ‘s companionship, their social support scores are higher, including the accessibility of medical services (availability of early dementia screening, quality of professional care), social participation opportunities (frequency of community activities, intergenerational support networks), and diverse cultural and recreational environments, which provide them with more opportunities for social interaction and cognitive stimulation (10). To a certain extent, it can buffer the negative impact of empty nest status on cognitive function. The interaction mechanism and multi-dimensional influencing factors need to be further explored.

Influenced by China’s dual social structure, there are marked differences in medical service resource allocation and health service levels between urban and rural areas, and the issue of urban–rural health equity has attracted great attention from scholars and governments at home and abroad (11). Relevant policies and guidelines clearly state the need to vigorously promote the equalization of basic public services in the health sector, ensure the public welfare nature of basic medical and health services, gradually narrow the gaps in basic health services and health levels between urban and rural areas, different regions, and different population groups, achieve universal health coverage, and promote social equity (12). However, given the limited social public resources and differences in socioeconomic status, individual physiological conditions, and accessibility to medical services, rural residents have significant disadvantages in health compared to urban residents (13). This disadvantage is likely to accumulate over the individual lifecycle into old age, further exacerbating the inherent differences in older adult health between urban and rural areas and leading to obvious imbalances in health status and medical service provision among older adult populations in urban and rural areas (14).

With the growing demand for older adult care services and the gradual weakening of the family support function, there is a huge tension between the imprecise supply of preventive healthcare services for cognitive impairment. Influenced by lifestyle, the prevalence of chronic diseases among the older adult is gradually increasing, with a coexistence of multiple chronic conditions reaching 32.4–40.2% (15), and cognitive function is closely related to the number and types of chronic diseases (16). Although existing studies have paid attention to the cognitive status of the older adult and urban–rural differences, there is still a lack of clear conclusions regarding the specific factors contributing to cognitive differences between urban and rural areas and their degrees of contribution, especially in-depth discussions targeting the vulnerable group of empty nest older adult. Therefore, this study aims to explore the impact of sociodemographic characteristics, chronic disease status, anxiety, depression, and cognitive function among Chinese older adult aged 65 and above, analyze urban–rural differences, and delve into the potential causes of such differences, providing scientific evidence and decision support for formulating effective prevention strategies for cognitive impairment and enhancing urban–rural medical equity.

## 2 Objects and methods

### 2.1 Study design and participants

This study employed a cross-sectional design, recruiting eligible empty nest older adult individuals from 35 cities and rural areas across 14 regions in Guangxi, China, between March and October 2023. Comprehensive health screenings and questionnaires were conducted. Inclusion criteria for empty nest older adult participants were: (1) age ≥65 years; (2) residence in the local area for ≥6 months; (3) no children or children not cohabiting; (4) conscious and possessing communication abilities. Exclusion criteria were: (1) those with severe mental or cognitive disorders; (2) those who did not complete all surveys and requested withdrawal. All data were assessed and collected by uniformly trained healthcare professionals. A total of 2,126 questionnaires were collected. With individuals excluded due to incomplete data on chronic conditions, depressive symptoms, anxious symptoms, or cognitive function. Ultimately, health information from 2083 empty nest older adult individuals was successfully compiled, and the recovery rate was 97.98%. All collected information was entered into a database using an electronic questionnaire platform. This study protocol was approved by the Ethics Review Committee of the Second Affiliated Hospital of Guangxi Medical University (Approval Number: 2023-KY0905), and all participants signed informed consent forms after being fully informed.

### 2.2 Data measurement

#### 2.2.1 Measurement of the risk of cognitive impairment

We utilized the 8-item Subjective Cognitive Decline (AD-8) questionnaire, leveraging feedback from the primary caregivers of older adult individuals, to assess their cognitive status. AD-8 serves as a convenient and efficient screening tool, demonstrating high sensitivity to early cognitive changes in various common dementias (17). The questionnaire covers multiple dimensions including judgment, daily living skills, personal initiative, orientation, and memory, with each item assignable a value of 0 or 1. Therefore, the total score range of AD-8 is 0 to 8, with higher scores indicating more severe cognitive impairment, and a score ≥2 suggesting a risk of cognitive impairment (18).

#### 2.2.2 Measurement of chronic diseases

Information on chronic conditions was collected by asking the following question: “Have you ever been diagnosed by a doctor or other medical professional with any of the following chronic conditions?” Participants were required to respond with “yes” or “no.” The list of chronic conditions was designed based on the International Classification of Diseases, 10th Revision (ICD-10). Combining previous epidemiological research and prevalence data (19), we selected the following 20 chronic diseases and symptoms: hypertension, heart disease/coronary heart disease, diabetes, cerebrovascular disease, chronic bronchitis, cancer, kidney disease, liver disease, gastroenteritis or other digestive diseases, tuberculosis, rheumatoid arthritis/arthritis, cervical/lumbar spine disease, reproductive system disease, prostate disease, urinary system disease, glaucoma or cataracts, osteoporosis, emotional and mental problems, neurological diseases, and deafness. older adult individuals diagnosed with any of these conditions in a hospital were considered to have a chronic condition.

#### 2.2.3 Measurement of depressive symptoms

We adopted the PHQ-9 questionnaire to assess depressive symptoms among participants, which is a valid scale based on the Diagnostic and Statistical Manual of Mental Disorders (DSM)-V for evaluating depressive symptoms over the past 2 weeks. The questionnaire contains 9 items, with each item rated on a four-point scale from “0” (not at all) to “3” (nearly every day), resulting in a total score range of 0 to 27. A score ≥5 indicates the presence of depressive symptoms (20).

#### 2.2.4 Measurement of anxiety symptoms

The Generalized Anxiety Disorder-7 (GAD-7) scale, developed by Spitzer et al., is used for screening and assessing the severity of generalized anxiety. It consists of 7 items designed to inquire about the frequency of disturbance experienced by respondents over the past 2 weeks in 7 areas, including feeling nervous and worried. The total score range is 0 to 21, with a score ≥5 indicating the presence of anxious symptoms (21).

### 2.3 Statistical analysis

Descriptive statistics and factorial analysis were performed using SPSS 23.0 software. Chi-square tests and logistic multivariate regression were used to explore differences in the risk of cognitive impairment among older adult individuals with different sociodemographic characteristics. The nonlinear Oaxaca-Blinder decomposition method in Stata 18 software was used to analyze urban–rural differences in cognitive function among empty nest older adult. All significance tests were bilateral, with a test level of *α* = 0.05, and statistical significance was set at *p* < 0.05. The Blinder-Oaxaca decomposition method consists of two steps:

Step 1: Conduct group-specific regressions to establish separate regression models for cognitive function among urban and rural empty nest older adult individuals. Here, Group 1 denotes the urban group, while Group 2 denotes the rural group, with regressions performed separately for each group (Equations 1, 2).


(1) Y1=α1+β1X1

(2) Y2=α2+β2X2

Step 2: Decomposition of differences, decompose the differences in cognitive function between urban and rural empty nest older adult individuals to explore the contribution of endowment characteristics of urban and rural empty nest older adult to these differences (Equation 3).


(3) $$\begin{array}{l} R=Y1-\mathrm{Y2}=\alpha 1-\alpha 2+\beta 1X1-\beta 2\mathrm{X2}=\beta 2(X1-\mathrm{X2})\\ +[\mathrm{X1}(\beta 1-\beta 2)+(\alpha 1-\alpha 2)] \end{array}$$

After deformation, the formula is finally divided into two parts. The first half of β2 (X1−X2) is called interpretable difference (rationality difference or endowment difference), which comes from the difference of endowment characteristics between urban and rural empty nest older adult. The second half of X1(β1−β2) is called unexplainable difference (discriminatory difference or coefficient difference). This part of the difference cannot be explained by the endowment difference between the two sample groups, which is generally considered to be caused by discrimination. (α1−α2) is a constant term, indicating the effect of unobserved factors on the cognitive function of urban and rural empty nest older adult.

## 3 Results

### 3.1 Univariate analysis of basic characteristics and the risk of cognitive impairment among empty nest older adult

This study included a total of 2,083 empty nest older adult individuals, among which 997 were from urban areas with an average age of (74.19 ± 6.98) years and an average number of years of education at (6.57 ± 4.11) years; 1,086 were from rural areas with an average age of (75.20 ± 7.01) years and an average number of years of education at (3.67 ± 3.28) years. The proportion of the risk of cognitive impairment among rural empty nest older adult was 33.33%, higher than that among urban empty nest older adult (26.88%), with a statistically significant difference (χ^2^ = 10.259, *p* = 0.001). The results of univariate analysis showed that gender, age, education level, marital status, number of chronic diseases, hypertension, cerebrovascular disease, depression, and anxiety had an impact on the cognitive function of urban empty nest older adult, and the differences were statistically significant (*p* < 0.05). Gender, age, education level, marital status, number of chronic diseases, hypertension, osteoporosis, cervical and lumbar spine disease, rheumatoid arthritis, depression, and anxiety have an impact on the cognitive function of rural empty nest older adult, and the differences are statistically significant (*p* < 0.05, Table 1).

**Table 1:** Single factor analysis of basic information and the risk of cognitive impairment of empty nest older adult.

Variables	Urban *n* (%)			*χ^2^*	*p*	Rural *n* (%)			*χ^2^*	*p*
	Total 997	Normal 729 (73.12)	Cognitive impairment 268 (26.88)			Total 1,086	Normal 724 (66.67)	Cognitive impairment 362 (33.33)		
Gender										
Male	400	306 (76.5)	94 (23.5)	3.884	0.049	502	373 (74.30)	129 (25.70)	24.495	<0.001
Female	597	423 (70.85)	174 (29.15)			584	351 (60.10)	233 (39.90)		
Age										
65–70	380	314 (82.63)	66 (17.37)	53.181	<0.001	335	251 (74.93)	84 (25.07)	47.065	<0.001
71–79	398	294 (73.87)	104 (26.13)			456	323 (70.83)	133 (29.17)		
≥80	219	121 (55.25)	98 (44.75)			295	150 (50.85)	145 (49.15)		
Educational period										
0	151	86 (56.95)	65 (43.05)	34.100	<0.001	319	153 (47.96)	166 (52.04)	77.090	<0.001
1–6	352	247 (70.17)	105 (29.83)			576	415 (72.05)	161 (27.95)		
≥7	494	396 (79.84)	98 (20.16)			191	156 (81.68)	35 (18.32)		
Marital status										
Married	578	441 (76.30)	137 (23.70)	7.068	0.008	500	359 (71.80)	141 (28.20)	10.988	<0.001
No spouse	419	288 (68.74)	131 (31.26)			586	365 (62.29)	221 (37.71)		
Number of chronic diseases										
0	305	249 (81.64)	56 (18.36)	22.332	<0.001	354	276 (77.97)	78 (22.03)	54.766	<0.001
1	399	290 (72.68)	109 (27.32)			402	271 (67.41)	131 (32.59)		
2	174	114 (65.52)	60 (34.48)			209	120 (57.42)	89 (42.58)		
3	72	48 (66.67)	24 (33.33)			86	46 (53.49)	40 (46.51)		
≥4	47	28 (59.57)	19 (40.43)			35	11 (31.43)	24 (68.57)		
Types of chronic diseases										
Hypertension	395	273 (69.11)	122 (30.89)	5.340	0.021	371	225 (60.65)	146 (39.35)	9.189	0.002
Diabetes	123	83 (67.48)	40 (32.52)	2.271	0.132	74	45 (60.81)	29 (39.19)	1.225	0.268
Cerebrovascular disease	54	30 (55.56)	24 (44.44)	8.961	0.003	72	43 (59.72)	29 (40.28)	1.673	0.196
Osteoporosis	43	28 (65.12)	15 (34.88)	1.464	0.226	63	28 (44.44)	35 (55.56)	14.862	<0.001
Cervical and lumbar spondylosis	133	92 (69.17)	41 (30.83)	1.216	0.270	169	104 (61.54)	65 (38.46)	2.369	0.024
Rheumatoid arthritis	110	73 (66.36)	37 (33.64)	2.871	0.090	102	55 (53.92)	47 (46.08)	8.229	0.004
Depressive state										
Depression	101	51 (50.50)	50 (49.50)	29.268	<0.001	140	51 (36.43)	89 (63.57)	66.128	<0.001
Normal	896	678 (75.67)	218 (24.33)			946	673 (71.14)	273 (28.86)		
Anxiety state										
Anxiety	61	28 (45.90)	33 (54.10)	24.490	<0.001	78	28 (35.90)	50 (64.10)	35.802	<0.001
Normal	936	701 (74.89)	235 (25.11)			1,008	696 (69.05)	312 (30.95)		

### 3.2 Logistic regression analysis of factors influencing cognitive function among empty- nest older adult in China

Table 2 includes variables that were statistically significant in the univariate analysis, and Logistic regression analysis was conducted separately for factors influencing cognitive function among empty nest older adult in different residential areas. The results showed that age, years of education, and depression were statistically associated with cognitive function among urban empty nest older adult (*p* < 0.05). Age, years of education, number of chronic diseases, cervical and lumbar spine diseases, and depression were statistically associated with cognitive function among urban empty nest older adult (*p* < 0.05). Rural empty nest older adult demonstrated sensitivity to the number of chronic diseases and cervical and lumbar spine diseases.

**Table 2:** Logistic regression of factors influencing cognitive function in empty nest older adult.

Variables	Residence	*B*	Standard error	Wald	*p*	Exp (B)	95% CI
Gender	Urban	0.055	0.163	0.114	0.736	1.056	0.768–1.453
	Rural	0.129	0.155	0.691	0.406	1.138	0.839–1.543
Age	Urban	0.407	0.103	15.494	<0.001	1.502	1.227–1.840
	Rural	0.294	0.098	8.990	0.003	1.342	1.107–1.627
Educational period	Urban	−0.344	0.106	10.465	0.001	0.709	0.576–0.873
	Rural	−0.735	0.123	35.620	<0.001	0.479	0.377–0.610
Marital status	Urban	−0.017	0.159	0.012	0.914	0.983	0.720–1.342
	Rural	0.044	0.149	0.087	0.768	1.045	0.780–1.400
Number of chronic diseases	Urban	0.177	0.114	2.400	0.071	1.193	0.954–1.492
	Rural	0.455	0.110	17.103	<0.001	1.576	1.270–1.955
Hypertension	Urban	−0.067	0.192	0.121	0.728	0.935	0.643–1.362
	Rural	−0.140	0.186	0.566	0.452	0.869	0.604–1.252
Cerebrovascular disease	Urban	0.257	0.326	0.621	0.431	1.293	0.682–2.449
	Rural	−0.106	0.293	0.131	0.717	0.899	0.506–1.598
Osteoporosis	Urban	0.011	0.363	0.001	0.975	1.011	0.497–2.059
	Rural	0.215	0.317	0.458	0.498	1.240	0.666–2.308
Cervical and lumbar spondylosis	Urban	−0.040	0.247	0.026	0.873	0.961	0.592–1.561
	Rural	−0.419	0.222	3.553	0.045	1.658	1.426–2.017
Rheumatoid arthritis	Urban	0.050	0.261	0.037	0.847	1.052	0.631–1.753
	Rural	−0.037	0.260	0.021	0.886	0.963	0.579–1.604
Depression	Urban	0.588	0.282	4.330	0.037	1.800	1.035–3.131
	Rural	0.960	0.249	14.885	<0.001	2.612	1.604–4.253
Anxiety	Urban	0.429	0.355	1.454	0.228	1.535	0.765–3.081
	Rural	0.212	0.320	0.436	0.509	1.236	0.659–2.316
Constant	Urban	−1.243	0.489	6.460	0.011	0.288	
	Rural	−0.795	0.495	2.580	0.108	0.451	

### 3.3 Nonlinear Oaxaca-Blinder decomposition of cognitive function differences among rural empty nest older adult

The results in Table 3 indicate that the explainable portion of the difference in cognitive function between urban and rural empty nest older adult accounts for 52.36%, while the unexplainable portion accounts 47.64%. This suggests that during the sample survey period, both the coefficient effect (urban–rural difference) and the characteristic effect of the sampled population played significant roles. The unexplainable portion reflects the inherent attributes of urban and rural areas that amplify the differences in cognitive function between urban and rural empty nest older adult, while the explainable portion reflects that individual characteristics, on the whole, narrow the differences in cognitive function among empty nest older adult in different residential areas. The characteristic variables with statistical significance in the explainable portion, ranked by their contribution rates, are: education level (44.09%), number of chronic diseases (27.74%), depression (15.75%), osteoporosis (10.79%), and age (6.19%). This indicates that the differences in these factors between urban and rural areas contribute to widening the disparities in cognitive function among empty nest older adult in different residential areas. The remaining variables have no statistically significant effect on the differences in cognitive function among empty nest older adult in different residential areas.

**Table 3:** Nonlinear Oaxaca Blinder decomposition of cognitive function differences among empty nest older adult living in different habitats.

Variables	*β*	Contribution rate	Contribution ranking	*p*
Gender	0.08 (−0.00344 ~ 0.00371)	−0.14	12	0.939
Age	−2.03 (−0.01184 ~ −0.00021)	6.19	5	0.042
Educational period	−4.26 (−0.06270 ~ −0.02318)	44.09	1	<0.001
Marital status	−1.57 (−0.00918 ~ 0.00101)	4.19	6	0.117
Number of chronic diseases	−3.39 (−0.04262 ~ −0.01140)	27.74	2	0.001
Hypertension	1.18 (−0.00206 ~ 0.00838)	−3.24	7	0.236
Diabetes	−0.38 (−0.00135 ~ 0.00091)	0.23	10	0.704
Cerebrovascular disease	−0.17 (−0.00114 ~ 0.00096)	0.09	13	0.866
Osteoporosis	2.45 (0.00211 ~ 0.01890)	−10.79	4	0.014
Cervical and lumbar spondylosis	0.51 (−0.00410 ~ 0.00699)	−1.48	8	0.61
Rheumatoid arthritis	−0.31 (−0.00739 ~ 0.00539)	1.03	9	0.758
Depression	2.41 (0.00284 ~ 0.02789)	−15.75	3	0.016
Anxiety	−0.26 (−0.00174 ~ 0.00132)	0.21	11	0.793
Explainable part	−3.68 (−0.07818 ~ −0.02379)	52.36		0
Unexplainable part	−2.05 (−0.09076 ~ −0.00203)	47.64		0.04

## 4 Discussion

The results of this study indicate significant urban–rural differences in cognitive function among empty nest older adult, with a lower proportion of normal cognitive function among rural empty nest older adult compared to their urban counterparts. According to CLHLS data analysis, rural empty nest older adult generally score lower than urban empty nest older adult in cognitive dimensions such as time-place orientation and reading comprehension (22). And further research has pointed out that there are differences in the rate of cognitive decline between urban and rural older adult (23), which may be related to the scarcity of medical resources, inadequate health interventions, and educational disparities in rural areas (24). Additionally, this study found that rural empty nest older adult exhibit sensitivity to the number of chronic diseases and cervical and lumbar spine diseases. Research shows that the coexistence rate of multiple chronic diseases among rural older adult over 70 years old (68.4%) is 17.2 percentage points higher than that of urban older adult (51.2%) (25). Among them, levels of chronic inflammatory factors such as CRP and IL-6 are associated with cognitive decline. Inflammation accelerates neuronal damage by activating microglia in the brain and producing proinflammatory mediators, leading to the decline in cognitive function (26). The World Health Organization (WHO)'s Global Report on Aging and Health 2021 points out that rural older adult populations in developing countries suffer from an average of 3.2 chronic diseases, significantly higher than the 2.1 chronic diseases among urban older adult populations, with musculoskeletal diseases accounting for up to 47% (27). This is closely related to long-term engagement in high-intensity physical labor in rural areas. For example, Sibson et al. (28) found through biomechanical modeling that the spinal load of agricultural workers is 3.9 times that of urban office workers. Meanwhile, cervical and lumbar spine diseases affect the attitudes and emotions of older adult individuals in their daily lives, leading to feelings of depression and low mood, which further restricts the social interactions of rural empty nest older adult. This makes it difficult for them to obtain spiritual comfort through social interactions, potentially leading to rapid cognitive decline (29).

Further Blinder-Oaxaca analysis results indicate that education level is the most significant factor influencing the urban–rural difference in cognitive function, accounting for 44.09%. In other words, if rural residents achieved the same level of education as urban residents, 44.09% of the urban–rural difference could be reduced. Education is considered a positive factor in delaying the decline of cognitive function. Those who have not received education often struggle to access pensions, owned housing, medical resources, and self-regulation in old age, and they are unable to independently understand changes in their health, which in turn affects cognitive function (30). The average years of education among rural empty nest older adult (3.67 years) is only 56% of that among urban older adult (6.57 years). Research shows that insufficient cognitive reserve due to lack of formal education is an independent factor associated with cognitive decline (31). Secondly, the prevalence of depression among rural empty nest older adult is significantly higher than that in urban areas. Depression, as a catalyzing factor, can lead to HPA axis dysfunction, which accelerates the atrophy of the hippocampus and is closely related to cognition (32). Additionally, the urban–rural difference in osteoporosis exacerbates physical limitations. Studies have found that rural women have significantly lower bone density than urban women, and this, combined with the pain-sleep deprivation pathway, increases the risk of cognitive decline by 1.9 times (33). Finally, as people age, their physical functions decline, neural functions diminish, and the risk of cognitive impairment increases. This age-cumulative effect is more pronounced in rural areas with inadequate medical resources and is not affected by factors such as social environment and psychological support (34). Therefore, for the empty nest older adult with low education levels, it is necessary to establish health records for older adult people, provide mental health services to alleviate feelings of loneliness and anxiety, and encourage the participation of rural older adult in social activities to enhance their social support networks, thereby strengthening their sense of social belonging and promoting psychological and mental health.

Furthermore, the differences that remain unexplained and yet statistically significant suggest that some variations in the utilization of physical examination services cannot be accounted for by differences in variable characteristic levels alone. This implies that, even when urban and rural older adult individuals have the same values for the independent variables, differences in urban–rural attributes can still lead to differences in the utilization of physical examination services. These unexplained differences may stem from variations in coefficients among different variables. For instance, even with the same level of education, cognitive function can still vary between urban and rural areas due to factors such as marital status, hypertension, and anxiety. Research has shown that the lack of marital support reduces social participation among older adult individuals, indicating that social connections and emotional exchanges remain effective in improving cognitive function among empty nest older adult, who tend to have a stronger dependence on emotional support (35). The impact of hypertension and its management on cognition has also been confirmed by numerous studies. Data suggests that medication adherence among rural hypertensive patients is 22% lower than that in urban areas (36), and poorly controlled hypertension can increase the volume of white matter hyperintensities in the brain, leading to impaired cognitive function (37). The urban–rural difference in anxiety exacerbates the risk of cognitive impairment through HPA axis activation, with chronic anxiety resulting in an annual atrophy rate of 0.9% in the gray matter volume of the prefrontal cortex (38).

Lastly, this study has certain limitations. Firstly, the data used in the study is cross-sectional, which allows for the exploration of correlations between the risk of cognitive impairment and various factors among urban and rural empty nest older adult, but cannot establish causality. Secondly, the variables used in the decomposition of differences mainly include social characteristics and chronic conditions of the older adult, with less consideration given to social and environmental factors that affect cognition. Therefore, in the future, prospective cohort studies or long-term monitoring data can be used to include social and medical environmental factors that affect the cognitive function of empty-nest older adult, and to analyze the impact of urban and rural potential category heterogeneity on cognitive function in terms of the accessibility of older adult care services, spatial activity, lifestyle and economic conditions of the older adult, so as to identify the differences between urban and rural empty-nest older adult in different dimensions of the risk of cognitive impairment.

## 5 Conclusion

There still exists structural inequality in cognitive health risks during the aging process in China, with a lower proportion of normal cognitive function among rural empty nest older adult compared to their urban counterparts. Additionally, individual differences in education level, number of chronic conditions, depression, cervical and lumbar spine diseases, and age between urban and rural empty nest older adult exacerbate disparities in cognitive function. In the future, it is necessary to establish a differentiated health intervention system for urban and rural areas. Cities should focus on psychological intervention and the development of community support networks, while rural areas urgently need to strengthen primary care management of chronic conditions and enhance cognitive reserve through educational compensation mechanisms (such as older adult learning centers), in order to systematically narrow the urban–rural gap in cognitive health.

## Data Availability

The raw data supporting the conclusions of this article will be made available by the authors, without undue reservation.
